# Cause and Effect Analysis between Influencing Factors Related to Environmental Conditions, Hunting and Handling Practices and the Initial Microbial Load of Game Carcasses

**DOI:** 10.3390/foods11223726

**Published:** 2022-11-20

**Authors:** Birsen Korkmaz, Denny Maaz, Felix Reich, Carl Gremse, Annina Haase, Rafael H. Mateus-Vargas, Anneluise Mader, Ingo Rottenberger, Helmut A. Schafft, Niels Bandick, Karsten Nöckler, Thomas Alter, Monika Lahrssen-Wiederholt, Julia Steinhoff-Wagner

**Affiliations:** 1 German Federal Institute for Risk Assessment (BfR), 10589 Berlin, Germany; 2 Berlin Brandenburg State Laboratory, Gerhard-Neumann-Straße 2, 15236 Frankfurt (Oder), Germany; 3 Institute of Food Safety and Food Hygiene, Center for Veterinary Public Health, Department of Veterinary Medicine, Freie Universität Berlin, Königsweg 69, 14163 Berlin, Germany; 4 TUM School of Life Sciences Weihenstephan, Animal Nutrition and Metabolism, Technical University of Munich, Liesel-Beckmann-Str. 2, 85354 Freising-Weihenstephan, Germany

**Keywords:** microbial growth, *Enterobacteriaceae*, *Escherichia coli*, body weight, ambient temperature, shooting accuracy, evisceration method, meat hygiene, FMEA

## Abstract

Environmental, hunting and handling factors affect the microbial load of hunted game and the resulting meat products. The aim of this study was to systematically investigate the influence of several factors on the initial microbial load (IML) of game carcasses during the early hunting chain. Eviscerated roe deer body cavities (*n* = 24) were investigated in terms of total viable count and the levels of *Pseudomonas* spp., *Lactobacillus* spp., *Enterobacteriaceae* and *Escherichia coli* (*E. coli*). Furthermore, a risk analysis based on the obtained original IML data, literature search and a Failure Mode and Effects Analysis (FMEA) was performed. The IML could be explained in a regression model by factors including the higher body weight (BW), damaged gastrointestinal tract by the shot, ambient temperature or rain. The levels of *Lactobacillus* spp. (*p* = 0.0472), *Enterobacteriaceae* (*p* = 0.0070) and *E. coli* (*p* = 0.0015) were lower on the belly flap surface when gloves were used during evisceration. The literature search revealed that studies examining influencing factors (IF) on the IML of game carcasses found contradictory effects of the comparable IF on IML. Potential handling failures may lead to a higher IML of game carcasses during the early hunting chain ranked by FMEA. Several handling practices for game carcasses are recommended, such as ensuring efficient cooling of heavier BW carcasses to limit bacterial growth or eviscerating heavier carcasses before lighter ones.

## 1. Introduction

Game meat is becoming increasingly popular due to its beneficial nutritional [1,2,3], ethical and sustainability aspects [4]. Since game animals inhabit various territories with different environmental conditions, the initial microbial load (IML) of game meat is influenced by the circumstances before and after the animal is hunted [5,6,7,8,9,10]. For example, hunting can be performed using different hunting methods, which may result in varying IML [11,12]. The stages of a hunt include observation, killing, salvage (recovery from the place of killing), evisceration and transport of the game in the field to a collection point or direct to the game-handling establishment or another storage location. Other steps may be implemented, such as bleeding of the carcass before evisceration [5,6,11]. Besides the hunting method and several published factors such as the ambient temperature on the hunting day [13,14,15], other factors may have a high impact on the IML of game carcasses. One example is the killing process itself. Several studies have reported that the shooting accuracy affects IML [16,17], while other studies have found no influence of this factor [5,18]. In Germany, hunters must pass an examination that tests knowledge and skills such as shooting, game hygiene and other topics before they are allowed to hunt. Subsequently, however, they are not normally required to demonstrate regular practice or further training. When killing game animals, hunters aim to shoot the game animal in the heart. Other factors related to the killing process that have been discussed but not confirmed as influencing bacterial load include ammunition construction [5], the shooting or escape distance [16] of the game. It is important to examine the conditions of the early steps of the hunting chain in their entirety and their effect on IML to improve game meat quality through handling recommendations or prevention strategies when handling game carcasses.

According to Regulation (EC) No. 178/2002, only safe products may be placed on the market in the European Union [19]. Obtaining and producing safe food with limited equipment and in non-standardized conditions, such as natural environments, is a challenge for game meat hygiene. In this regard, quality assurance and management concepts such as Hazard Analysis Critical Control Point (HACCP) in accordance with European Regulation (EC) 852/2004 [20] could help food business operators in producing safe food. However, this concept is hard to apply to the hunting chain. This is because the HACCP analysis begins with the identification of potential hazards to consumer health along a standardized production process, but no standardized process exists for obtaining game carcasses as primary products in the field. Each hunt is unique due to animal-related parameters, environmental conditions and the killing process; also, the hunting and handling practices are variable. For example, during drive hunts in Germany, the time that elapses between killing and eviscerating the game could be very different [5]. In this matter, a Failure Mode and Effects Analysis (FMEA) can be used to generate a preliminary impression of the potential failures in handling game carcasses during the hunting chain and to estimate their impact on the IML of game carcasses. FMEA is a powerful method for identifying critical points in a process and preventing failures [21] that may result in a high IML of game carcasses and meat.

A high IML of carcasses is a potential risk for low-quality game meat [22]. Nevertheless, there are still no microbial limits for game meat as exist for meat obtained from livestock [23]. Data on bacterial loads in game carcasses have been published, e.g., for environmental bacteria, fecal bacteria [5,6] and/or pathogens [15] under a variety of environmental and hunting conditions and using different sampling methods and matrices, depending on the objective of each study. This complicates the comparability of the microbial data and the specification of a generally valid microbial limit or warning value for the different animal species. However, Paulsen et al. [24] propose a total bacterial count of 10^6^ CFU/cm² as a provisional warning limit for roe deer (*Capreolus capreolus*) carcasses based on a veterinary post-mortem inspection of “conspicuous” roe deer carcasses.

In the present study, animal-related parameters, environmental conditions, factors of the killing process as well as hunting and handling practices were investigated to identify which parameters most strongly affect the IML of hunted and eviscerated roe deer carcasses from Brandenburg, Germany. The magnitude of each identified influencing factor (IF) on IML was assessed in this study in the context of a statistical risk analysis, literature search and an FMEA. Based on the IFs that can lead to higher IMLs, potential handling failures were identified. Conversely, recommendations for the handling of game carcasses were provided on the basis of IFs that may lead to a reduction in IML.

## 2. Materials and Methods

### 2.1. Collection of Data on Animal-Related, Environmental, Ammunition and Shooting, as Well as Hunting and Handling Parameters

This study was conducted complying with ethical standards, the data privacy agreement of the German Federal Institute for Risk Assessment, and with federal and institutional animal use guidelines. Roe deer (*n* = 24) were shot within the framework of wildlife management [25] and for human consumption in the hunting season 2020–2021 (*n* = 19) and 2021–2022 (*n* = 5) by several hunters on 12 hunting estates in Brandenburg, Germany. Roe deer carcasses were obtained during the annual drive hunt-season (autumn and winter season in the Northern hemisphere) at comparably low ambient temperatures organized by the German Federal Forestry Service at hunting districts administered by the German Federal Institute for Real Estate (BImA) or at hunting districts of the state forest of Brandenburg. Data on the hunted roe deer were recorded for the early steps of hunting chain and contained information on sex, body weight (BW) after evisceration, weather conditions (especially ambient temperature and rain on the day of hunt), ammunition used, duration between killing and evisceration, technique of evisceration, use of gloves during evisceration and presence of visible soiling on the roe deer body cavity with gastrointestinal contents. Parts of this study with a total of 23 roe deer carcasses were previously published as a set of 19 roe deer from the season 2020–2021 by Korkmaz et al. [26]. The data for four carcasses from that study were statistically incomplete, so five additional roe deer carcasses were sampled in the hunting season 2021–2022 including all required data to reach a comparable sample size.

### 2.2. Sampling and Microbial Investigation of Swab Samples from Roe Deer

Swab samples according to ISO 17604:2015 were taken from the meat surface of the belly flaps (*M. obliquus internus abdominis*) and the fillets (*M. psoas major*) with a moistened swab (3.8 × 7.6 cm; 3M Sponge-Stick; Mercateo Deutschland AG, Munich, Germany) followed by a dry swab (16 × 152 mm, Greiner Bio-One cotton swab; Altmann Analytik GmbH & Co. KG, Munich, Germany). Sampling of the belly flap and fillet surface was executed in the center of the indicated region with an area of 50 cm² or 20 cm², respectively. Swab samples were rinsed with 90 mL diluent (Maximum Recovery Diluent for microbiology; Merck, Darmstadt, Germany) according to ISO 6887-1:2017 in a bag mixer (BagMixer^®^ 400, step 3, 120 s; Interscience, Saint Nom, France).

The total viable count (DIN ISO 4833-2:2014, Plate Count Agar; Carl Roth, Karlsruhe, Germany), the levels of *Pseudomonas* spp. (specifications of the manufacturer, Pseudomonas/Aeromonas selective agar; Sigma-Aldrich, Darmstadt, Germany), *Lactobacillus* spp. (DIN 10109:2017, de Man Rogasa and Sharpe agar; Carl Roth, Karlsruhe, Germany) and *Enterobacteriaceae* (DIN 10164:2019, Violet Red Bile Dextrose agar; Merck, Darmstadt, Germany) were analyzed by the spread plate method. After aerobic incubation at 30 °C for 72 h or anaerobic incubation at 37 °C for 24 h for *Enterobacteriaceae*, counts of the respective bacterial groups were calculated. Presumptive colonies of *Pseudomonas* spp. were confirmed by positive oxidase testing (ROTITEST^®^ Oxidase strips; Carl Roth, Karlsruhe, Germany). The level of *Escherichia coli* (*E. coli*, DIN ISO 16649-2:2010, Tryptone Bile X-glucuronide Agar; Carl Roth, Karlsruhe, Germany), was determined by the pour plate method after aerobic incubation at 44 °C for 24 h. The counts of bacteria examined were given in log_10_ CFU/cm².

### 2.3. Statistical Risk Analysis

Linear regressions with backward variable elimination were performed to identify potential factors affecting IML as target variables. The target variables for every regression included the total viable counts, the counts of *Pseudomonas* spp., *Lactobacillus* spp., *Enterobacteriaceae* and *E. coli* on the meat surface of the belly flap and fillet, respectively. The normality of the target variable distributions was examined with the Shapiro–Wilk Test after logarithmic transformation. Potential factors affecting IML included BW after evisceration, sex of roe deer carcasses, ambient temperature and occurrence of rain on the hunting day, ammunition construction used with assigned impact energy at 100 m distance, shooting accuracy, shooting distance between hunter and roe deer, escape distance of roe deer, duration between killing and evisceration, evisceration technique and position of carcass during this process, usage of gloves, as well as presence of visible soiling of the roe deer body cavity with gastrointestinal content as independent factors. All regressions were calculated in R Statistics (R-Version 4.1.2., R Core Team 2022) using the function “lm” (package stats). Backward variable elimination was performed using the “step” function (package stats). Variables were excluded stepwise until the Akaike Information Criterion (AIC) could not be improved further. All of the resulting “best models” for every regression (every combination of bacterial group and sampled muscle) revealed *p*-values ≤ 0.05 in the F-statistic. In order to quantify the magnitude of the effects of the resulting IF in the “best models” on the IML, Rate Ratios (RR) were determined by calculating the exponential function of the model estimates. A RR corresponds to a factor by which, according to the model, the IML (log_10_ CFU/cm²) increases (if RR > 1) or decreases (if RR < 1) if a specific level of an IF (e.g., animal sex: female) occurs in comparison to a reference level (e.g., male), or if an increase in a metric IF occurs (e.g., +1 kg body weight). To make the effect statements more tangible, RRs to ambient temperature, shooting and escape distance, duration between killing and evisceration were calculated for increments of ten. The data on, e.g., animal-related parameters or the IML examined of the carcasses were summarized descriptively using SPSS Software version 26 (IBM, Ehningen, Germany). Heat maps and stacked bar graphs were created in GraphPad Prism 9.3.1 (GraphPad Software, San Diego, CA, USA).

### 2.4. Literature Search of Factors Affecting the Initial Microbial Load of Game Carcasses Based on Previously Published Data in Original Research Articles

The literature was screened on 23 May 2022 for previously reported IFs on IMLs of game carcasses. The search was conducted using Google Scholar with the English search terms “weight bacteria game meat” or “carcass microbial contamination game” without any restriction. The articles to be screened were selected according to the relevance of their titles. Additional articles cited by the initially screened articles were considered and checked for relevance. Results on the identified IFs and on the bacterial load of game carcasses were classified by animal species, sample size, significance and bacterial group studied.

### 2.5. Failure Mode and Effects Analysis Based on Authors’ Expertise or on a Defined Stepwise Search

**Figure 1:**
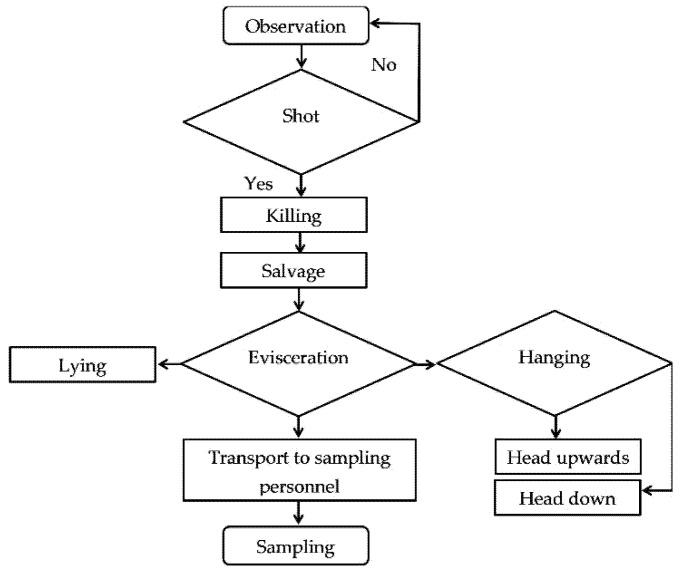
Flowchart for the investigated early steps of the hunting chain from observation of the living game animal to the sampling of the carcasses.

A flowchart of obtaining game carcasses along the early steps of the hunting chain was created and used for the two FMEA approaches: one based on the authors’ expertise and one based on a defined stepwise search (Figure 1). In this study, the assessed part of the hunting chain started with game observation and ended with the collection of samples from the killed and eviscerated carcasses in the field. Potential failures during handling of game carcass were identified based on the results of this study and the literature search.

To assess the impact of IFs on IML of game carcasses during each step of the hunting chain, a Risk Priority Number (RPN) was calculated. The RPNs for each possible failure was calculated by multiplication of the estimated values from 1 to 5 for the probability of occurrence (O), the significance (S) and the probability of detection (D). The calculated RPN can range from 1 to 125, with the failure or risk becoming less acceptable as the RPN increases. In this study, the risk of adverse impact of handling failures on IML was classified as low risk with an RPN of <19, medium risk with 29 > RPN > 20 and high risk with an RPN ≥30 based on the FMEA performed.

There is no single standard for the rating scale of an FMEA. However, the scale of 1 to 5 is preferred because it allows for the easy interpretation of a possible failure during a process [21]. Furthermore, the weighting of the rating scale is process-dependent and related to a meaningful class formation. In this study, O, S and D were each divided into five classes of the rating scales (Table 1). Two FMEAs were performed based on either the authors’ expertise or a defined stepwise search, described in Section 2.5.2. The RPNs of both FMEAs were graphically compared for the same defined handling failures in stacking bars. Variability of the given RPNs by experts were presented as boxplots. The illustrations were created in GraphPad Prism 9.3.1 (GraphPad Software, San Diego, CA, USA).

#### 2.5.1. Failure Mode and Effects Analysis Based on Authors’ Expertise

The consultation of the authors for the FMEA was performed by using a survey. The possible handling failures were formulated openly, so that the experts had to prioritize based on their own experiences. This was executed to ensure that the evaluation is based on the aspect that seems the most critical for the respective author and covers as many sources of failures as possible while obtaining game carcasses in a common context. Therefore, the authors ranked RPNs using multiple scenarios and viewpoints and considering various potential IFs.

#### 2.5.2. Failure Mode and Effects Analysis Based on Defined Stepwise Search

Since the FMEA based on the authors’ expertise included personal bias, it was complemented by an FMEA based on a defined stepwise search of scientific evidence. Therefore, values of O, S and D were first classified based on the effects of IFs on IML determined by linear regression and RRs in the context of a risk analysis in this study. When the classification of factors affecting IML could not be explained by the results of original IML data, other original research articles were reviewed for evidence as a second step. This was the case when data for the relevant IF were not obtained in our own study. As a third step, when there was a lack of published evidence (either in this or in another study), the classification was based on experience reported by hunters in grey literature.

## 3. Results

### 3.1. Animal-Related Parameters, Environmental Factors, Ammunition and Shooting, as Well as Hunting and Handling Parameters

**Figure 2:**
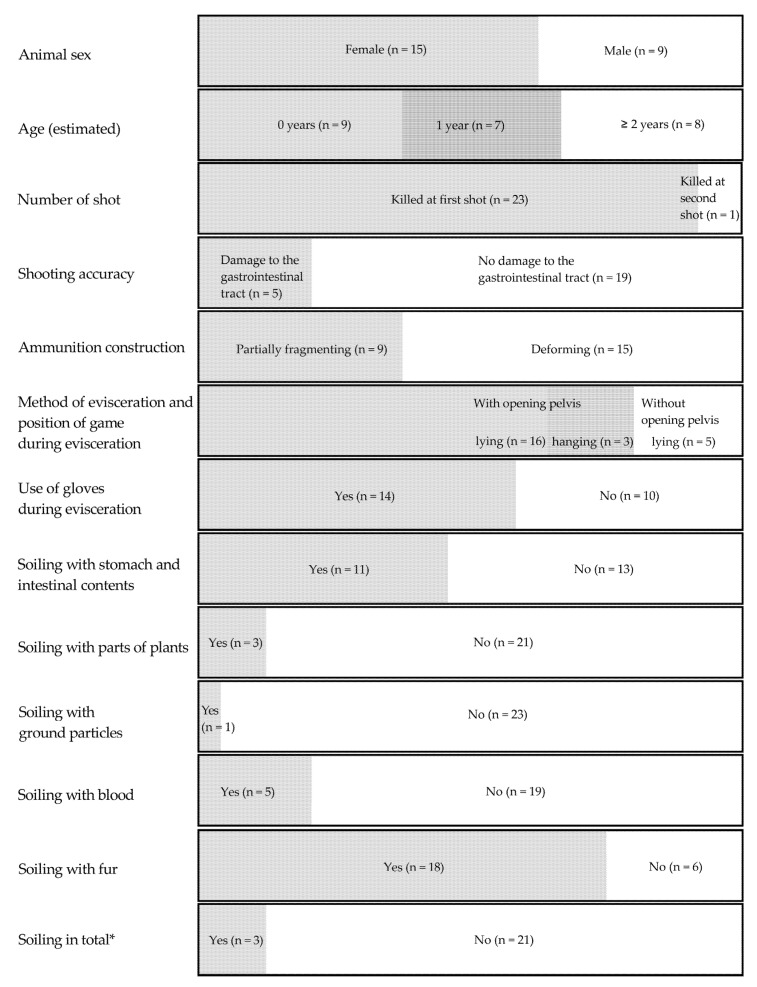
Animal-related parameters and parameters representing shooting-related factors, handling of roe deer carcasses as well as visual evaluation of the body cavity. * Soiling in total was classified as “no” if no visible contamination was present in the body cavity and “yes” if one or more types of contamination were present in the body cavity.

Freshly eviscerated roe deer carcasses (*n* = 24) were examined from 2020 to 2022 by taking swab samples of the belly flap (*n* = 24) and fillet (*n* = 23) surfaces on hunting day. Roe deer carcasses were obtained on six rainy hunting days (*n* = 13) and eight dry hunting days (*n* = 11). Of the roe deer carcasses, 21% showed damage to the gastrointestinal tract. However, visible soiling of the body cavity by gastrointestinal contents after the handover appeared in 46% of the carcasses (Figure 2).

The BW of the roe deer carcasses after evisceration varied from 8.4 to 18.2 kg (median 13.5 kg, 95% confidence interval (CI) 11.0–15.1 kg). The ambient temperature measured during the sampling of the carcasses ranged from 0 to 13 °C (median 5 °C, 95% CI 2.0–10.3 °C). Based on manufacturer’s specifications, the impact energy of ammunition at 100 m distance ranged from 2358 to 3484 J (median 2765 J, 95% CI 2759–3247 J). The time from killing the roe deer until evisceration ranged from 5 to 240 min (median 148 min, 95% CI 78–180 min). The shooting distance between the hunter and the roe deer was estimated to be up to 60 m (median 40 m, 95% CI 20–50 m). Half of the sampled roe deer were killed by the shot directly in place, the other half after an escape distance between 2 and 50 m.

### 3.2. Initial Microbial Load of Meat Surfaces of the Body Cavity

The total viable count mean and standard deviation (SD) were similar on both meat surfaces: 3.8 ± 1.0 log_10_ CFU/cm² on the belly flap surface and 4.0 ± 1.1 log_10_ CFU/cm² on the fillet surface. The counts of *Pseudomonas* spp., *Enterobacteriaceae* and *E. coli* were also similar in both sample matrices. The levels of *Lactobacillus* spp. were higher in the fillet than in the belly flap (Table 2).

### 3.3. Factors Influencing the Initial Microbial Load of Freshly Eviscerated Roe Deer Carcasses

**Figure 3:**
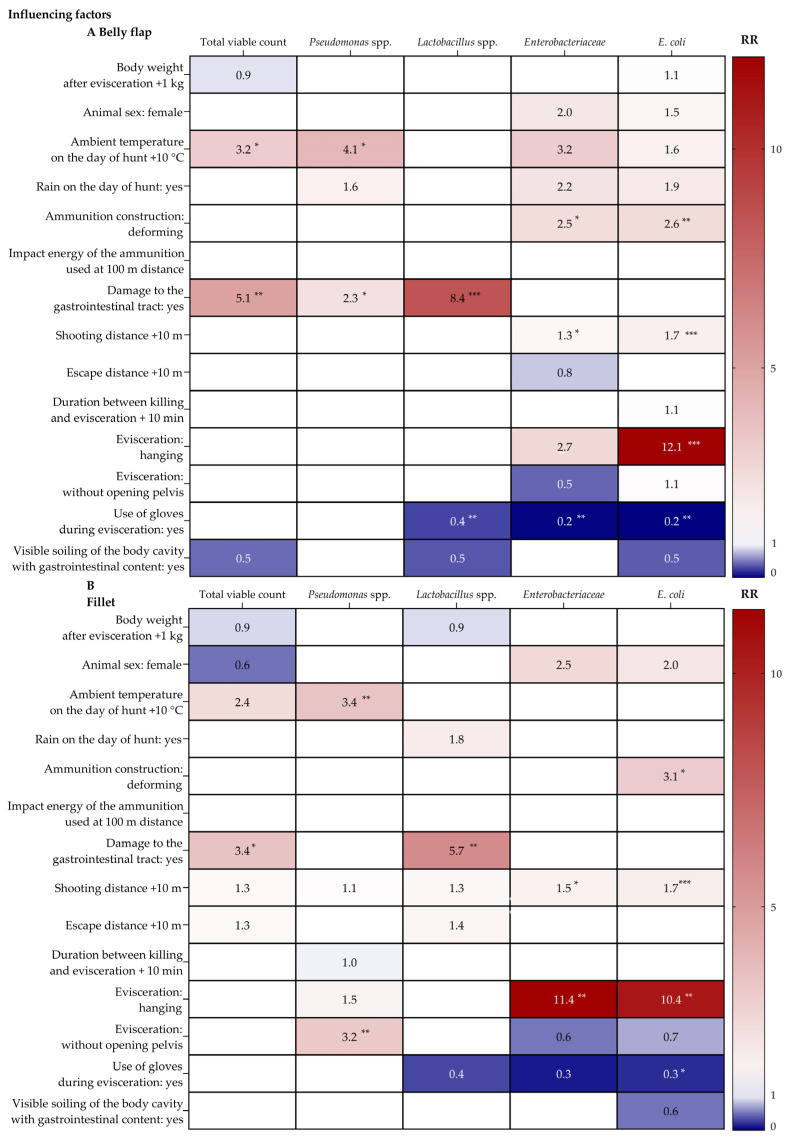
Heat maps for influencing factors (IFs) affecting initial microbial load (IML; total viable colony count, *Pseudomonas* spp., *Lactobacillus* spp., *Enterobacteriaceae*, *E. coli*) of roe deer belly flap ((**A**), *n* = 24) and fillet ((**B**), *n* = 23) with resulting Rate Ratios (RRs) shown in each cell. The heat maps have been created using the RRs of variables identified as IFs by linear regression with backward selection. An RR of 1 were presented as empty cells and means no effect. Significance levels of RRs were highlighted by stars (* *p* < 0.05, ** *p* < 0.01, *** *p* < 0.001). To make the effect statements more tangible, RRs were calculated for ambient temperature, shooting distance, escape distance and duration between killing and evisceration in increments of ten.

IFs were defined in this study as parameters that could have an impact on IML, are measurable or categorizable or could be managed in hunting practice. Ambient temperature (0–13 °C) had an effect on the bacterial load of carcasses. Keeping other variables constant, an increase in ambient temperature may result in a 3.2-fold higher total viable count and a 4.1-fold higher *Pseudomonas* spp. count in the belly flap (95% CI 1.3–7.7 log_10_ CFU/cm²; 95% CI 1.9–8.9 log_10_ CFU/cm²) and a 3.4-fold higher *Pseudomonas* spp. count in the fillet (95% CI 1.5–7.7 log_10_ CFU/cm², Figure 3). Damage to the gastrointestinal tract resulted in a higher bacterial load by 5.1 for total viable count, by 2.3 for *Pseudomonas* spp. and by 8.4 for *Lactobacillus* spp. in the belly flap (95% CI 2.1–12.4 log_10_ CFU/cm²; 95% CI 1.1–5.0 log_10_ CFU/cm²; 95% CI 2.9–24.2 log_10_ CFU/cm²) compared to carcasses shot without gastrointestinal damage. Likewise, the total viable count may be 3.4-fold and the *Lactobacillus* spp. counts 5.7-fold higher in the fillet (95% CI 1.1–10.1; 95% CI 1.8–18.1 log_10_ CFU/cm²).

Furthermore, carcasses eviscerated with opening in a hanging position had higher values of *E. coli* in the belly flap (RR = 12.1, 95% CI 4.6–31.6 log_10_ CFU/cm²) and higher values of *Enterobacteriaceae* and *E. coli* in the fillet (RR = 11.4, 95% CI 1.4–90.1 log_10_ CFU/cm²; RR = 10.4, 95% CI 2.4–44.4 log_10_ CFU/cm²) than carcasses eviscerated lying on the ground (*n* = 16). Carcasses eviscerated without opening the pelvis (*n* = 5) had higher levels of *Pseudomonas* spp. in the fillet (RR = 3.2, 95% CI 1.5–6.6 log_10_ CFU/cm²) than carcasses with the pelvis opened (*n* = 16).

When hunters eviscerated carcasses using gloves, levels of *Lactobacillus* spp., *Enterobacteriaceae* and *E. coli* in the belly flap (RR = 0.4, 95% CI 0.2–0.99 log_10_ CFU/cm²; RR = 0.2, 95% CI 0.1–0.5 log_10_ CFU/cm²; RR = 0.2, 95% CI 0.1–0.6 log_10_ CFU/cm²) and the values of *E. coli* in the fillet (RR = 0.3, 95% CI 0.1–0.9 log_10_ CFU/cm²) were lower than in carcasses eviscerated without using gloves.

Compared to partially fragmenting bullets, use of deforming bullets caused higher initial levels for *Enterobacteriaceae* and *E. coli* in the belly flap (RR = 2.5, 95% CI 1.0–6.1 log_10_ CFU/cm²; RR = 2.6, 95% CI 1.4–4.7 log_10_ CFU/cm²) and higher levels of *E. coli* in the fillet (RR = 3.1, 95% CI 1.3–7.6 log_10_ CFU/cm²).

When the shooting distance between the hunter and the roe deer increased, the values of *Enterobacteriaceae* and *E. coli* in the belly flap (RR = 1.4, 95% CI 1.0–1.8 log_10_ CFU/cm²; RR = 1.7, 95% CI 1.4–2.0 log_10_ CFU/cm²) and in the fillet (RR = 1.5, 95% CI 1.1–2.2 log_10_ CFU/cm²; RR = 1.7, 95% CI 1.3–2.2 log_10_ CFU/cm²) were elevated (Figure 3; Tables S1 and S2 (Supplementary Materials)).

### 3.4. Factors Influencing the Initial Microbial Load of Game Carcasses Based on a Literature Search

**Figure 4:**
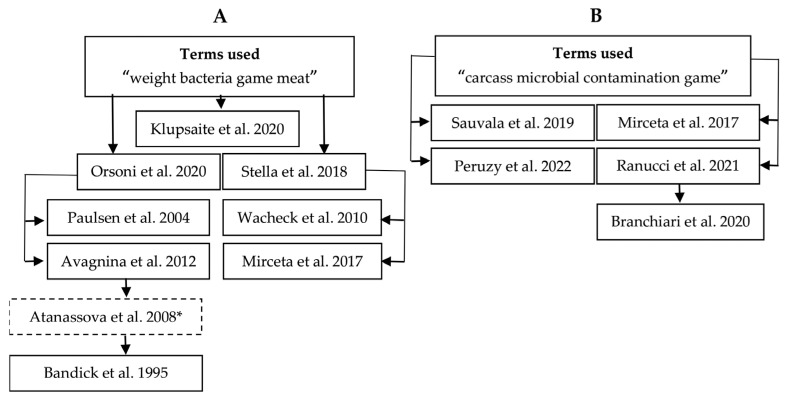
Original research articles found through the literature search in Google Scholar with the terms “weight bacteria game meat” (**A**) [3,7,9,14,15,16,18,27,28] and “carcass microbial contamination game” (**B**) [5,6,8,9,13]. Arrows represent direct hits of the search term or the primary reference that cited the related study. * The discontinuous frame indicates a reference that was excluded by the described criteria, but served as a lead for another reference.

During the literature search, 34 articles with relevant titles on microbial investigation of game carcasses were reviewed twice using the selected terms in Google Scholar (Figure 4). Of these, 13 articles were considered in more detail as they contained results on IF on the bacterial load of game carcasses (Table 3, Table 4 and Table 5). Articles with a focus on the IML and with using a convincing statistical method were included in the stepwise FMEA, whereas e.g., descriptive papers were only used as an alternative groundwork for the discussion of observations.

### 3.5. Failure Mode and Effects Analysis

**Figure 5:**
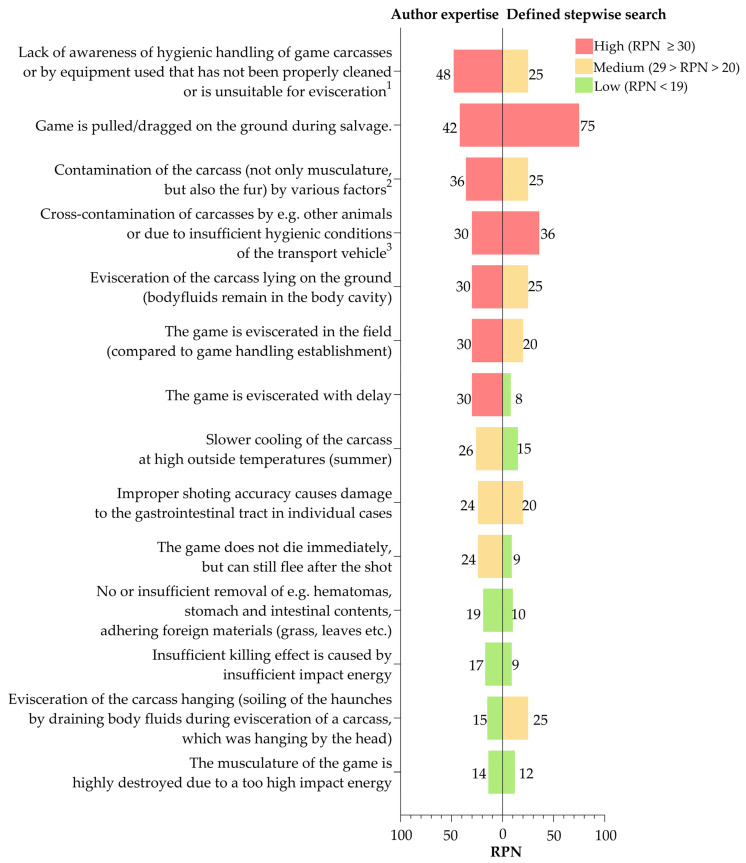
Risk Priority Numbers (RPNs) were compared using the authors’ expertise and a defined stepwise search. This search was conducted based on the results of this study, other published original research articles or when there was no published evidence on reported hunters’ experiences from the grey literature. The subjects are shown as presented in the (expert) assessment including the footnotes for further specification: (1) contamination of carcass by, e.g., unwashed hands in the absence of running water or improper handling with gloves or unclean or blunt knives; (2) e.g., rain, grass, leaves, surface water, etc. on the ground when the tarpaulin is not in use or when the stomach and intestinal tract of the game is damaged during evisceration and the contents contaminate the carcass; (3) e.g., stacking or too close placement of several killed animals on a transport vehicle that is contaminated with soil, leaves, blood residues from eviscerated carcasses.

The FMEA based on the authors’ expertise and the defined stepwise search identified the shooting/killing, salvage, evisceration and transport steps as having the greatest potential for failure. Handling failures that can affect carcass IML are e.g., lack of awareness of hygienic handling of game carcasses (contamination of carcass by, e.g., unwashed hands in the absence of running water or improper handling with gloves or by using improperly cleaned or unsuitable equipment, e.g., unclean or blunt knives); pulling/dragging the game on the ground during salvage; contamination of the carcass (not only musculature, but also the fur) by various factors, e.g., rain, grass, leaves, surface water, etc., on the ground when the tarpaulin is not in use or when the stomach and intestinal tract of the game is damaged during evisceration and the contents contaminate the carcass; cross-contamination of carcasses by e.g., other animals (stacking or too close placement of several killed animals on a transport vehicle) or due to insufficient hygienic conditions of the transport vehicle (e.g., soil, leaves, blood residues from eviscerated carcasses); evisceration of the carcass lying on the ground (body fluids remain in the body cavity); evisceration of the game in the field; and the game is eviscerated with delay (Figure 5).

**Figure 6:**
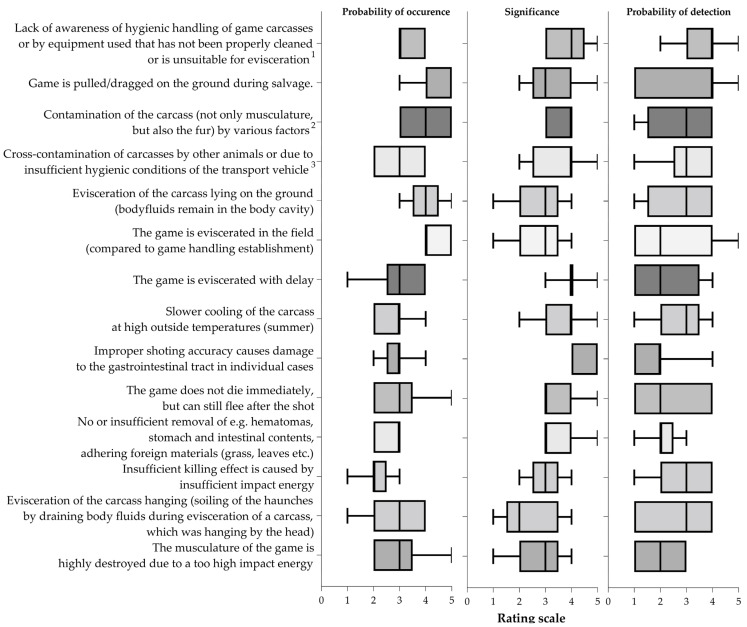
Boxplots show the variability of the probability of occurrence, significance and probability of detection rankings with minimum and maximum values for Failure and Mode and Effect analysis based on authors’ expertise. The subjects of the assessments including the footnotes for further specification: (1) contamination of carcass by, e.g., unwashed hands in the absence of running water or improper handling with gloves or unclean or blunt knives; (2) e.g., rain, grass, leaves, surface water, etc. on the ground when the tarpaulin is not in use or when the stomach and intestinal tract of the game are damaged during evisceration and the contents contaminate the carcass; (3) e.g., stacking or too close placement of several killed animals on a transport vehicle that is contaminated with soil, leaves, blood residues from eviscerated carcasses.

The RPNs of the FMEA based on the authors’ expertise and defined stepwise search differed mainly due to the different definitions used to evaluate the probability of detection. High risk RPNs were obtained more often in the FMEA based on authors´ expertise than using the defined stepwise search. However, there is a similarity in the assessed RPNs of handling failures (Figure 5), i.e., cross-contamination of carcasses by other animals (high risk RPN), improper shooting accuracy causing damage to the gastrointestinal tract (medium risk RPN) or the musculature of the game animal being highly damaged due to too high impact energy (low risk RPN). The assessment of some other RPNs varied between the experts. An impact of delayed evisceration on IML was ranked similarly by the experts (Figure 6).

## 4. Discussion

The IML of game carcasses is affected by IFs and provides an indication of the microbial quality of the resulting food product. The IML of hunted carcasses is higher than that of livestock animal carcasses slaughtered under controlled conditions; additionally, game meat obtained in the field is generally more likely to show sensory deviations, faster spoilage and consequently have a reduced shelf-life [31]. However, if appropriate hygienic measures are taken, such as the use of gloves when eviscerating carcasses, game meat with improved microbial quality can be obtained even under field conditions, as shown in this study. Therefore, the initial processing of game meat is very important. Although the bacterial load of consumed game products can be influenced by many other factors later in the value chain, the focus in this study on the early steps of game meat harvesting was made since bacterial growth is exponential and this period is the most lacking in controlled conditions. This study highlights IFs on the IML of game carcasses processed under field conditions. Due to various IFs at a hunt covering animal-related parameters, environmental factors, hunting and handling practices, the IML of carcasses may vary between animals even during the same hunt. This results in data that can appear very complex between studies and are rarely comparable. In addition, it seemed that still some potentially important IFs have not yet been supported by evidence. Therefore, based on risk analysis of original IML data and a literature search, the present study identified factors that can be described numerically or by categorization and that may have a significant impact on the IML of carcasses. With this study, more evidence is available for the IFs on IML: ambient temperature; the presence of rain during the hunt; the shooting and escape distance of the game or the carcass’s BW. The magnitude of these IFs on IML of game carcasses was determined based on a holistic approach combining RRs and FMEA to mirror the relevance of each factor to potential handling failures during the early hunting chain.

The results of this study were discussed along the timeline of the steps of the hunting chain (Figure 1). Hunting begins with the observation of the living game to assess the game animals’ appearance and behavior and thus their health condition. Furthermore, the game animals were classified by the species, sex, age or BW. Stella et al. [15] have reported higher bacterial loads for male wild boar carcasses and Branciari et al. [5] found no significant influence of the sex on roe deer carcasses. Another IF could be the animal species, because the IML of ruminants are different from those of wild boars [18]. Wild boars, as monogastric animals, have different gastrointestinal anatomy and microbiome compared to roe deer as ruminants. The results across species were nevertheless used since sample matrices, methods, locations or bacterial groups examined seemed comparable to the present study. Furthermore, the age of the animals was not determined as an appropriate IF, neither in this study based on a risk analysis of the original IML data nor in the study by Stella et al. [15]. The hunters estimated the age classes of animals based on the visible body condition of the animals, e.g., the shape of the antlers of the male animal. Since the age class estimation is imprecise and the reported age depends on the individual experience of the hunter, this parameter seemed unsuitable to be used as an IF for the IML up to now. However, the impact of age class on IML of carcasses could be interacting with the possible effects of sex or BW of the sampled carcasses on the IML, which could be investigated in future research projects with more valid age information and a higher sample size.

Based on the risk analysis of the original IML data from roe deer carcasses in the present study, the BW of the animals was identified as an IF on the total viable count and is in accordance with the findings by Branciari et al. [5]. Stella et al. [15] were able to determine the influence of wild boar BW only for *Enterobacteriaceae* levels, although total bacterial counts were also examined. Carcass BW can be measured and thus, is less susceptible to reporting bias. Based on a literature search [5,7,8,15], higher BW may result in a higher IML. For example, carcass handling of heavier individuals may impair proper hygienic handling and could result in higher bacterial counts [32]. This could be improved, for instance, by having a second person to assist with the handling of heavier carcasses.

The next step in the hunting chain is to shoot and kill the game, which represents a very individual scenario for each animal, resulting in the differences in identifying the potential impact factor on the IML as stated above. The parameters used in literature to describe the shooting and killing process qualitatively are the shooting accuracy [5,6], number of shots [6], impact energy or caliber of ammunition used [5,6], pre-mortem stress of game [33], shooting distance [11] or escape distance [16,34]. However, it has hardly been confirmed if and how these conditions influence the bacterial load. Based on our risk analysis, the ammunition construction, impact energy of the ammunition, improper shooting accuracy, shooting and escape distance contributed as IFs on IML and might result in gastrointestinal tract damage or the delayed death of the game animal. However, the effects of these factors described on the killing process of animals depend mainly on the decisions made by the hunter prior to the shot. Since the effects of these qualitative IFs on the IML are difficult to interpret, two FMEA were applied. The FMEA based on the authors´ expertise assessed the escape of shot game that do not die immediately as a medium-risk failure for higher IML (averaged RPN = 24) while FMEA based on a defined stepwise search assessed escape as a low risk (RPN = 9). A higher evaluation of the significance and the probability of detection by the experts led to this difference. Some experts commented that they also considered other IFs in this scenario, such as an incorrect shot accuracy or longer time until chilling. On the contrary, the RPN calculation based on a stepwise literature search was restricted to only one defined IF.

After the game animal is killed, it is salvaged from the place of killing. The hunter could carry the carcass or drag the carcass on the ground, which usually depends on the game’s BW. Since the samples in this study were taken after the carcasses had already been salvaged, eviscerated and transported within the field, the impairments by the salvage practices only were impossible to identify. In addition, information on the impact of salvaging on IML in original research articles is also lacking. However, using FMEA based on the authors’ expertise and the defined stepwise search, dragging carcasses on the ground during salvage was ranked as a high-risk handling failure due to the probability of occurrence and significance. This handling failure harbors the risk that during dragging, the fur of the carcass might be contaminated with soil or bacteria, which could be transferred to the meat during evisceration or skinning. Bacterial contamination, e.g., of carcass fur, is a major source of cross-contamination on the meat surface [22]. The probability of detection of cross-contamination was ranked comparably high in FMEA, based on the authors’ expertise and the defined stepwise search.

Based on the total cause-and-effect analysis, the evisceration process includes several factors that may contribute to a higher IML of carcasses due to handling practice. The place of evisceration was identified as an IF based on the authors’ expertise. Depending on the hunting method, environmental circumstances and the shortest possible duration between killing and evisceration, hunters have to decide whether they eviscerate killed game directly at the salvage location, after transport to a collection point or at a game chamber. The roe deer carcasses sampled in this study at drive hunts in Brandenburg were eviscerated on location according to the instructions of the organizer of the hunt. Exposure to environmental conditions may affect the microbial condition of the carcass more frequently, such as the presence of rain. In particular, wet fur can make hygienic handling more difficult. The presence of rain was identified as an IF that can lead to higher IML in this study based on the risk analysis of original data, as was previously reported by Ranucci et al. [8] for wild boar carcasses. On rainy hunting days, it might be more beneficial for lower cross-contamination to transport the game carcass to a place protected from rain before evisceration.

Based on the risk analysis of original IML data, the evisceration technique used and position of roe deer carcass during evisceration were identified as additional IFs. Evisceration can be performed either with or without opening the pelvis on a carcass lying on the ground [9] or hanging from, e.g., a wild gallows [26]. Unexpectedly in this study, the IML of roe deer carcasses eviscerated hanging by the hind legs (*n* = 3) was higher than the IML of carcasses eviscerated lying on the ground (*n* = 16) with an opening in the pelvis. In contrast, Mirceta et al. [9] found higher bacterial loads in carcasses that were eviscerated lying under field conditions than in carcasses eviscerated hanging in a game handling establishment. However, opening the pelvis seemed to create a larger surface area in the body cavity that can become contaminated. Performing evisceration without opening the pelvis, the body cavity remains protected from surface contamination; however, this also seems to delay the cooling compared to carcasses with an open pelvis. This could promote the bacterial growth, especially at higher ambient temperatures [6,15,30,35]. In the current case, sampling was performed during the autumn and winter season in the Northern hemisphere at comparatively low ambient temperatures. Based on the findings of this study, it could also be beneficial for the microbial quality of carcasses to eviscerate heavier carcasses faster than carcasses with low BW and to open the pelvis of all carcasses. This is due to a potential interaction between the slower chill time of heavier carcasses and the ambient temperature, which was determined within the fitting regression model. However, the meat surface needs to be protected from contamination as much as possible during further handling.

Carcasses with damaged gastrointestinal tracts by improper shooting are known to show higher IML as confirmed by the risk analysis of the original IML data and the literature search [28]. In many cases, the microflora of the gastrointestinal content has a big impact on the IML [36]. In this study, the *Lactobacillus* spp. counts were higher in the belly flap and the fillet samples in roe deer carcasses killed with damage to the gastrointestinal tract.

The use of gloves during evisceration was queried. Based on the risk analysis of original IML data, lower bacterial counts were found when gloves were used than when they were not. Therefore, gloves can be used as a hygiene measure to obtain carcasses with a lower IML besides their use as a personal protection measure, e.g., possible infection with hepatitis E [37]. Beyond the direct effect of using gloves during carcass evisceration, lower IML of carcasses handled with gloves might also represent an indirect effect of the hunter’s awareness regarding hygiene measures in general. For example, Mirceta et al. [9] reported higher bacterial counts when untrained hunters eviscerated wild boar carcasses than trained hunters [9]. Furthermore, the lack of awareness of the hygienic handling of game carcasses has been determined as a main handling failure based on the FMEA.

The sampling of roe deer carcasses occurred in this study after the carcasses were transported within the field and handed over from the hunters to the sampling personnel. Based on FMEA, taking into account the collective transport of several carcasses at the same time, this transport step was assessed as a handling failure with a high risk of obtaining carcasses with a higher IML. Stacking of multiple animals should be avoided to reduce contamination and delayed cooling, as described before.

This study identified several IFs on IML in the early processing of the game meat chain with a holistic approach. Some factors are extremely difficult to identify because they appear rarely or irregularly in practice. Besides this, other methodological challenges can arise if different studies used other hunting practices or definitions for the same IFs. That is why, in this study, the quantitative original research results regarding factors influencing IML and the associated possible handling failures were combined with two FMEAs. Both FMEAs showed the highest variation in RPN due to the rating of the probability of detection of bacterial effects. This might reflect the fact that the IML is not visually detectable and can also be altered at the following steps of the hunting chain.

The most relevant handling failures are:

1. Lack of awareness of hygienic handling of game carcasses;
2. Pulling/dragging of carcass on the ground;
3. Contamination of the carcass (not only musculature, but also the fur);
4. Cross-contamination of carcasses during transport by e.g., other animal carcasses, delayed chilling or due to insufficient hygienic conditions of the transport vehicle;
5. Evisceration of the game in the field even if there is a possibility to eviscerate the game immediately in a game handling establishment;
6. Delay in the evisceration of the carcass.

## 5. Conclusions

This study identified factors that may influence the IML during the harvest of game carcasses using data on IML collected from roe deer carcasses as original research and using a literature search. In addition, the magnitude of these IFs on IML of game carcasses was estimated. Potential handling failures and recommendations during the hunting chain were investigated more closely based on the risk analysis of the original data, literature search and FMEA. This combined approach allows for the provision of some recommendations to persons who obtain game carcasses in the field for human consumption and thus participate in the first part of the supply chain for game meat. Visual cleanliness of carcasses does not have to be related to a low bacterial load. This underlines the significance of sensitizing and training the hunters on the importance of their practical contribution to lower the microbial load of game meat.

The study results for handling game carcasses support existing European regulations during harvest and highlight some new aspects, which are summarized hereinafter.

1. Hunters should be trained regarding hygiene including personal protection;
2. Contact of the carcass with the ground and other environmental factors should be reduced, as much as possible;
3. Game carcasses should be eviscerated without delay in a weather-protected place;
4. After the evisceration process of the carcass, the meat surface should be protected from cross-contamination as much as possible during further handling;
5. Special effort should be taken to keep the time after evisceration as short as possible to ensure effective chilling;
6. Multiple carcasses should be transported separately from each other.

## Figures and Tables

**Table 1:** Rating scales used in Failure Mode and Effects Analysis (FMEA) to classify the probability of occurrence, significance and probability of detection to assess the impact of handling failures during the hunting chain on the initial microbial load (IML) of game carcasses.

Classes of the Rating Scale	Rating Scale
**Probability of occurrence**	
1	Very unlikely to occur in the hunting practice
2	Unlikely to occur in the hunting practice
3	Possible to occur in the hunting practice
4	Likely to occur in the hunting practice
5	Very likely to occur in the hunting practice
**Significance**	
1	Very unlikely to have an impact on IML (very low probability of contamination and distribution of bacteria on/in the carcass) *
2	Unlikely to have an impact on IML (low probability of contamination and distribution of bacteria on/in the carcass) *
3	An impact on the IML is possible (contamination and distribution of bacteria on/in the carcass is probable) *
4	Likely to have an impact on IML (high probability of contamination and distribution of/in bacteria on the carcass) *
5	Very likely to have an impact on IML (very high probability of contamination and distribution of/in bacteria on the carcass) *
**Probability of detection**	
1	Detection of failure is very likely
2	Detection of failure is likely
3	Detection of failure is possible
4	Detection of failure is unlikely
5	Detection of failure is very unlikely

* For classification of significance on the IML, bacteria were assumed to have been transferred or distributed by contact or through animal metabolism.

**Table 2:** Mean, standard deviation (SD) and 95% confidence intervals (95% CI) of the initial microbial load on the meat surface of belly flap (*n* = 24) and fillet (*n* = 23) of roe deer carcasses.

Initial Microbial Load, log_10_ CFU/cm²	*n*	Mean	SD	95% CI
**Belly flap**				
Total viable count	24	3.8	1.0	3.4–4.3
*Pseudomonas* spp.	24	2.6	0.8	2.3–3.0
*Lactobacillus* spp.	24	2.4	1.3	1.8–3.0
*Enterobacteriaceae*	24	1.6	1.2	1.1–2.1
*E. coli*	24	1.0	1.2	0.5–1.5
**Fillet**				
Total viable count	23	4.0	1.1	3.5–4.5
*Pseudomonas* spp.	23	2.6	0.9	2.2–3.0
*Lactobacillus* spp.	23	3.1	1.4	2.5–3.7
*Enterobacteriaceae*	23	1.8	1.6	1.1–2.5
*E. coli*	23	1.3	1.4	0.7–2.0

**Table 3:** Environmental factors that may influence the initial microbial load of game carcasses including animal species, sample size, *p*-value and bacterial group examined, reported in the original research articles.

Influencing Factor	Animal Species	*n*	Bacterial Group	Significant Effect	*p*-Value	Reference
Ambient temperature	Moose/ White-tailed deer	100/ 100	Mesophilic aerobic bacteria	Yes	0.023	[13]
	Roe deer	64	Aerobic colony count	No	0.963	[5]
	Ungulates ‡	50	Total aerobic count	Yes	<0.05	[14]
	Wild boar	36	Mesophilic bacteria	No	>0.05	[6]
	Wild boar	120	Aerobic colony count	Yes	<0.05	[8]
	Wild boar	62	Total viable count	Yes	<0.01	[15]
	Moose/ White-tailed deer	100/ 100	*Enterobacteriaceae*	Yes	0.003	[13]
	Roe deer	64	*Enterobacteriaceae*	Yes	0.012	[5]
	Ungulates ‡	50	*Enterobacteriaceae*	Yes	<0.05	[14]
	Wild boar	36	*Enterobacteriaceae*	No	>0.05	[6]
	Wild boar	120	*Enterobacteriaceae*	Yes	<0.05	[8]
	Moose/ White-tailed deer	100/ 100	*E. coli*	Yes	0.011	[13]
	Wild boar	36	*E. coli*	Yes	<0.05	[6]
	Wild boar	62	Pathogens ***	No	-	[15]
	Wild boar	62	*Listeria* spp.	Yes	<0.05	[15]
Rain on the day of hunt	Wild boar	120	Aerobic colony count	No	>0.05	[8]
	Wild boar	120	*Enterobacteriaceae*	No	>0.05	[8]
	Ungulates ‡	50	Total aerobic count	Yes	<0.05	[14]
	Roe deer	119	*Enterobacteriaceae*	No	-	[29]
	Ungulates ‡	50	*Enterobacteriaceae*	Yes	<0.05	[14]

‡ 25 red deer, 18 roe deer, 3 chamois, 1 mouflon, 3 wild boar. *** *Campylobacter* spp., Salmonella and *L. monocytogenes;* - indicates lack of specified *p*-value.

**Table 4:** Animal-related factors that may influence the initial microbial load of game carcasses including animal species, sample size, *p*-value and bacterial group examined, reported in the original research articles.

Influencing Factor	Animal Species	*n*	Bacterial Group	Significant Effect	*p*-Value	Reference
Body weight after evisceration	Roe deer •	64	Aerobic colony	Yes	-	[5]
	Wild boar	36	Mesophilic bacteria	No	>0.05	[6]
	Wild boar	37	Aerobic colony count	Yes	0.014	[7]
	Wild boar	120	Aerobic colony count	Yes	<0.05	[8]
	Wild boar	62	Total viable count	No	-	[15]
	Roe deer •	64	*Enterobacteriaceae*	No	-	[5]
	Wild boar	62	*Enterobacteriaceae*	Yes	0.03	[15]
	Wild boar	36	*Enterobacteriaceae*	No	>0.05	[6]
	Wild boar	37	*Enterobacteriaceae*	No	-	[7]
	Wild boar	120	*Enterobacteriaceae*	Yes	<0.05	[8]
	Wild boar	36	*E. coli*	No	>0.05	[6]
	Wild boar	62	*E. coli*	Yes	0.04	[15]
	Roe deer •	64	Pathogens *	No	-	[5]
	Wild boar	36	Pathogens **	No	>0.05	[6]
	Wild boar	62	Pathogens ***	No	-	[15]
	Wild boar	153	Pathogens ****	No	0.3071	[27]
Animal sex	Moose/ White-tailed deer	100/ 100	Mesophilic aerobic bacteria	No	0.06	[13]
	Wild boar	36	Mesophilic bacteria	No	>0.05	[6]
	Wild boar	120	Aerobic colony count	No	>0.05	[8]
	Wild boar	62	Total viable count	No	-	[15]
	Moose/ White-tailed deer	100/ 100	*Enterobacteriaceae*	No	0.20	[13]
	Wild boar	36	*Enterobacteriaceae*	No	>0.05	[6]
	Wild boar	120	*Enterobacteriaceae*	No	>0.05	[8]
	Wild boar	62	*Enterobacteriaceae*	Yes	0.02	[15]
	Moose/ White-tailed deer	100/ 100	*E. coli*	Yes	0.03	[13]
	Wild boar	36	*E. coli*	No	>0.05	[6]
	Wild boar	62	*E. coli*	Yes	<0.01	[15]
	Wild boar	36	Pathogens **	No	>0.05	[6]
	Wild boar	62	Pathogens ***	No	-	[15]

• Body weight before evisceration. * *Salmonella* spp., *L. monocytogenes*; ** *Salmonella* spp., *Yersinia enterocolitica*, *Campylobacter* spp. and pathogenic *E. coli*; *** *Campylobacter* spp., *Salmonella*, *Listeria* spp. and *L. monocytogenes*; **** *Salmonella* spp., *Yersinia enterocolitica*, *Yersinia pseudotuberculosis*, STEC, *L. monocytogenes*; - indicates lack of specified *p*-value.

**Table 5:** Ammunition and shooting, hunting and handling factors that may influence the initial microbial load of game carcasses including animal species, sample size, *p*-value and bacterial group examined, reported in the original research articles.

Influencing Factor	Animal Species	*n*	Bacterial Group	Significant Effect	*p*-Value	Reference
Ammunition construction	Roe deer	64	Aerobic colony count	No	0.969	[5]
	Roe deer	64	*Enterobacteriaceae*	No	0.641	[5]
Damage to the gastrointestinal tract	Moose/ White-tailed deer	100/ 100	Mesophilic aerobic bacteria	No	≥ 0.20	[13]
	Roe deer	50	Aerobic colony count	Yes	-	[16]
	Roe deer	78	Aerobic Viable Count	Yes	-	[18]
	Wild boar	47	Aerobic colony count	Yes	-	[16]
	Wild boar	72	Aerobic Viable Count	Yes	-	[18]
	Wild boar	36	Mesophilic bacteria	No	>0.05	[6]
	Wild boar	210	Aerobic colony counts	No	-	[9]
	Wild boar	125	Total Viable Count	No	-	[30]
	Moose/ White-tailed deer	100/ 100	*Enterobacteriaceae*	Yes	0.009	[13]
	Wild boar	36	*Enterobacteriaceae*	No	>0.05	[6]
	Wild boar	210	*Enterobacteriaceae*	No	-	[9]
	Wild boar	125	*Enterobacteriaceae*	No	-	[30]
	Moose/ White-tailed deer	100/ 100	*E. coli*	No	-	[13]
Escape distance	Roe deer	50	Aerobic colony count	No	-	[16]
	Wild boar	47	Aerobic colony count	No	-	[16]
Duration between killing and evisceration	Roe deer	64	Aerobic colony count	Yes	0.049	[5]
	Wild boar	36	Mesophilic bacteria	No	>0.05	[6]
	Wild boar	37	Aerobic colony count	No	-	[7]
	Wild boar	120	Aerobic colony count	No	0.565	[8]
	Roe deer	64	*Enterobacteriaceae*	No	0.840	[5]
	Wild boar	36	*Enterobacteriaceae*	No	>0.05	[6]
	Wild boar	37	*Enterobacteriaceae*	No	-	[7]
	Wild boar	120	*Enterobacteriaceae*	No	0.082	[8]
	Wild boar	36	*E. coli*	No	>0.05	[6]
Evisceration location: field vs. game-handling establishment	Wild boar	210	Aerobic colony counts	Yes	<0.05	[9]
	Wild boar	210	Aerobic colony counts	Yes	<0.05	[9]
Evisceration: hanging	Wild boar	210	Aerobic colony counts	Yes	<0.05	[9]
	Wild boar	210	*Enterobacteriaceae*	Yes	<0.05	[9]
Visible soiling of body cavity with gastrointestinal content	Roe deer	119	Aerobic mesophilic bacteria	No	-	[29]
	Ungulates ‡	50	Total aerobic count	Yes	<0.05	[14]
	Roe deer	119	*Enterobacteriaceae*	No	-	[29]
	Ungulates ‡	50	*Enterobacteriaceae*	Yes	<0.05	[14]

‡ 25 red deer, 18 roe deer, 3 chamois, 1 mouflon, 3 wild boar; - indicates lack of specified *p*-value.

## Data Availability

The data presented in this article are available from the corresponding author upon reasonable request.

## Supplementary Materials

The following supporting information can be downloaded at: https://www.mdpi.com/article/10.3390/foods11223726/s1, Table S1: Rate Ratios of variables identified as influencing factors of bacterial species in roe deer belly flap (*n* = 24) by linear regression with backward selection with 95% confidence intervals (95% CI) and *p*-values. Significance levels of Rate Ratios were highlighted by stars (* *p* < 0.05, ** *p* < 0.01, *** *p* < 0.001); Table S2: Rate Ratios of variables identified as influencing factors of bacterial species in roe deer fillet (*n* = 23) by linear regression with backward selection with 95% confidence intervals (95% CI) and *p*-values. Significance levels of Rate Ratios were highlighted by stars (* *p* < 0.05, ** *p* < 0.01, *** *p* < 0.001); Table S3: Evaluation of the extent of failure based on the Risk Priority Number (RPN) calculated by the FMEA based on defined stepwise search. Values of O, S and D were classified based on the effects of IF on IML determined by linear regression and RRs in this study. When the classification of factors affecting IML could not be explained by the results of this study, the original research articles based on the literature search were reviewed for evidence. As a last step, when there was a lack of published evidence, classification was based on experience reported by hunters; Table S4: Rating of probability of detection (D) for possible handling failures during game carcass obtaining based on defined stepwise search (part 1); Table S5: Rating of probability of detection (D) for possible handling failures during game carcass obtaining based on defined stepwise search (part 2).
