# Supplementary material for: Cause and Effect Analysis between Influencing Factors Related to Environmental Conditions, Hunting and Handling Practices and the Initial Microbial Load of Game Carcasses
Source: Foods. 2022 Nov 20;11(22):3726. doi: 10.3390/foods11223726 (PMC9689144; doi:10.3390/foods11223726)
Supplement: Supplementary file 1 [file foods-11-03726-s001.zip › foods-2002382-supplementary.pdf]

foods

MDPI

Table S1. Rate Ratios of variables identified as influencing factors of bacterial species in roe deer belly flap (*n* = 24) by linear regression with backward selection with 95% confidence intervals (95% CI) and *p*-values. Significance levels of Rate Ratios were highlighted by stars (**p* < 0.05,** *p*< 0 .01,*** *p* < 0.001)

Influencing factor	Bacterial species	Rate Ratio	95% CI	*p*-value
Body weight after evisceration +1 kg	Total viable count	0.9	0.8 – 1.0	0.1592
	E. coli	1.1	1.0 – 1.2	0.1975
Animal sex: female	*Enterobacteriaceae*	2.0	0.8 – 5.2	0.1316
	E. coli	1.5	0.8 – 2.7	0.1941
Ambient temperature on the day of hunt +10 °C	Total viable count	3.2	1.3 – 7.7	0.0128*
	Pseudomonas spp.	4.1	1.8 – 8.9	0.0013**
	Enterobacteriaceae	3.2	0.9 – 11. 6	0.0747
	E. coli	1.6	0.6 – 4.0	0.2847
Rain on the day of hunt: yes	*Pseudomonas* spp.	1.6	0.9 – 3.0	0.1209
	Enterobacteriaceae	2.2	0.9 – 5.5	0.0911
	E. coli	1.9	0.9 – 4.0	0.0759
Ammunition contraction: deforming	*Enterobacteriaceae**	2.5	1.0 – 6.1	0.0467
	*E. coli***	2.6	1.4 – 4.7	0.0059
Damage to the gastrointestinal tract: yes	Total viable count	5.1	2.1 – 12.3	0.0011**
	Pseudomonas spp.	2.3	1.1 – 5.0	0.0332*
	Lactobacillus spp.	8.4	2.9 – 24.2	0.0004***
Shooting distance +10 m	*Enterobacteriaceae*	1.3	1.0 – 1.8	0.0324*
	E. coli	1.7	1.4 – 2.0	0.0001***
Escape distance +10 m	*Enterobacteriaceae*	0.8	0.6 – 1.2	0.2825
Duration between killing and evisceration +10 min	*E. coli*	1.1	1.0 – 1.1	0.0604
Evisceration: hanging	*Enterobacteriaceae*	2.7	0.6 – 11.4	0.1692
	E. coli	12.1	4.6 – 31.6	0.0001***
Evisceration: without opening pelvis	*Enterobacteriaceae*	0.5	0.2 – 1.5	0.2214
	E. coli	1.1	0.5 – 2.5	0.7271
Use of gloves during evisceration: yes	*Lactobacillus* spp.	0.4	0.2 – 1.0	0.0472*
	Enterobacteriaceae	0.2	0.1 – 0.6	0.0070**
	E. coli	0.2	0.1 – 0.5	0.0015**
Visible soiling of body cavity with gastrointestinal content: yes	Total viable count	0.5	0.3 – 1.1	0.0843
	Lactobacillus spp.	0.5	0.2 – 1.2	0.1010
	E. coli	0.5	0.2 – 1.0	0.0604

Table S2. Rate Ratios of variables identified as influencing factors of bacterial species in roe deer fillet (n = 23) by linear regression with backward selection with 95% confidence intervals (95% CI) and p-values. Significance levels of Rate Ratios were highlighted by stars (*p < 0.05, **p < 0.01, ***p < 0.001)

Influencing factor	Bacterial species	Rate Ratio	95% CI	p-value
Body weight after evisceration +1 kg	Total viable count	0.9	0.7 – 1.1	0.1618
	Lactobacillus spp.	0.9	0.7 – 1.1	0.2206
Animal sex: female	Total viable count	0.6	0.2 – 1.4	0.1986
	Enterobacteriaceae	2.5	0.7 – 8.8	0.1376
	E. coli	2.0	0.8 – 4.8	0.1207
Ambient temperature on the day of hunt +10 °C	Total viable count	2.4	0.8 – 7.5	0.1113
	Pseudomonas spp.	3.4	1.5 – 7.7	0.0069**
Rain on the day of hunt: yes	*Lactobacillus* spp.	1.8	0.7 – 4.5	0.2021
Ammunition contraction: deforming	*E. coli*	3.1	1.3 – 7.6	0.0172*
Damage to the gastrointestinal tract: yes	Total viable count	3.4	1.1 – 10.1	0.0299*
	Lactobacillus spp.	5.7	1.8 – 18.1	0.0056**
Shooting distance +10 m	Total viable count	1.3	0.9 – 1.7	0.1054
	Pseudomonas spp.	1.1	0.9 – 1.4	0.1769
	Lactobacillus spp.	1.3	1.0 – 1.8	0.0894
	Enterobacteriaceae	1.5	1.1 – 2.2	0.0156*
	E. coli	1.7	1.3 – 2.2	0.0003***
Escape distance +10 m	Total viable count	1.3	0.9 – 1.8	0.1745
	Lactobacillus spp.	1.4	1.0 – 2.1	0.0769
Duration between killing and evisceration +10 min	*Pseudomonas* spp.	1.0	0.9 – 1.0	0.0691
Evisceration: hanging	*Pseudomonas* spp.	1.5	0.5 – 4.5	0.4869
	Enterobacteriaceae	11.4	1.4 – 90.1	0.0241**
	E. coli	10.4	2.4 – 44. 4	0.0037**
Evisceration: without opening pelvis	*Pseudomonas* spp.	3.2	1.5 – 6.6	0.0044**
	Enterobacteriaceae	0.6	0.1 – 2.3	0.4193
	E. coli	0.7	0.3 – 2.0	0.5362
Use of gloves during evisceration: yes	*Lactobacillus* spp.	0.4	0.2 – 1.1	0.0858
	Enterobacteriaceae	0.3	0.1 – 1.0	0.0545
	E. coli	0.3	0.1 – 0.9	0.0268*
Visible soiling of body cavity with gastrointestinal content: yes	*E. coli*	0.6	0.2 – 1.4	0.2056

Table S3. Evaluation of the extent of failure based on the Risk Priority Number (RPN) calculated by the FMEA based on defined stepwise search. Values of O, S and D were classified based on the effects of IF on IML determined by linear regression and RRs in this study. When the classification of factors affecting IML could not be explained by the results of this study, the original research articles based on the literature search were reviewed for evidence. As a last step, when there was a lack of published evidence, classification was based on experience reported by hunters

Step of hunting chain	Failure	O	S	D	RPN
Salvage	Game is pulled/dragged on the ground during salvage.	5	5	3	75
Transport	cross-contamination of carcasses by e.g. other animals (stacking or too close placement of several killed animals on a transport vehicle) or due to insufficient hygienic conditions of the transport vehicle (e.g. soil, leaves, blood residues from eviscerated carcasses).	3	3	4	36
Evisceration	Evisceration of the carcass lying on the ground (body fluids remain in the body cavity, when eviscerating the carcass lying on the ground).	5	5	1	25
Evisceration	Evisceration of the carcass hanging (soiling of the haunches by draining body fluids during evisceration of a carcass, which was hanging by the head).	5	5	1	25
Evisceration	Lack of awareness of hygienic handling of game carcasses (contamination of carcass by, e.g., unwashed hands in the absence of running water or improper handling with gloves or by equipment used that has not been properly cleaned or is unsuitable for evisceration, e.g., unclean or blunt knives).	5	5	1	25
Evisceration	Contamination of the carcass (not only musculature, but also the fur) by various factors, e.g. rain, grass, leaves, surface water, etc. on the ground when the tarpaulin is not in use or when the stomach and intestinal tract of the game is damaged during evisceration and the contents contaminate the carcass.	5	5	1	25
Shooting/killing	Improper shooting accuracy causes damage to the gastrointestinal tract in individual cases.	4	5	1	20
Evisceration	The game is eviscerated in the field (compared to game handling establishment).	4	5	1	20
Evisceration	Slower cooling of the carcass at high outside temperatures (summer)	3	5	1	15
Shooting/killing	The musculature of the game is highly destroyed due to a too high impact energy.	3	4	1	12
Evisceration	No or insufficient removal of e.g. hematomas, stomach and intestinal contents, adhering foreign materials (grass, leaves etc.)	2	5	1	10
Shooting/killing	Insufficient killing effect is caused by insufficient impact energy.	3	3	1	9
Shooting/killing	The game does not die immediately, but can still flee after the shot.	3	3	1	9
Evisceration	The game is eviscerated with delay.	2	4	1	8

Table S4. Rating of probability of detection (D) for possible handling failures during game carcass obtaining based on defined stepwise search (part 1)

Possible failure	Rating according to	Parameter used for rating of D	Rating scale of D
Improper shooting accuracy causes damage to the gastrointestinal tract in individual cases	Original IML data	Damage to gastrointestinal tract affect IML	1
Insufficient killing effect is caused by insufficient impact energy	Original IML data	Body weight and ammunition construction affect IML	1
The musculature of the game is highly destroyed due to a too high impact energy	Original IML data	Body weight and ammunition construction affect IML	1
The game does not die immediately, but can still flee after the shot	Original IML data	Escape distance were identified as influence factor on IML	1
The game is eviscerated in the field (compared to game handling establishment)	Literature research	Evisceration location, field vs. game handling establishment affect bacterial load, reported by Mirceta et al. [9]	1
The game is eviscerated with delay	Original IML data	Duration between killing and evisceration affect IML	1
Evisceration of the carcass lying on the ground (body fluids remain in the body cavity, when eviscerating the carcass lying on the ground)	Original IML data	Evisceration position of the carcass affect IML	1
Evisceration of the carcass hanging (soiling of the haunches by draining body fluids during evisceration of a carcass, which was hanging by the head)	Original IML data	Evisceration position of the carcass affect IML	1
Lack of awareness of hygienic handling of game carcasses (contamination of carcass by, e.g., unwashed hands in the absence of running water or improper handling with gloves or by equipment used that has not been properly cleaned or is unsuitable for evisceration, e.g., unclean or blunt knives).	Original IML data	Gloves worn during evisceration affect IML	1

Table S5. Rating of probability of detection (D) for possible handling failures during game carcass obtaining based on defined stepwise search (part 2)

Possible failure	Rating according to	Parameter used for rating of D	Rating scale of D
Contamination of the carcass (not only musculature, but also the fur) by various factors, e.g. rain, grass, leaves, surface water, etc. on the ground when the tarpaulin is not in use or when the stomach and intestinal tract of the game is damaged during evisceration and the contents contaminate the carcass.	Original IML data	Rain at hunting day affect IML	1
Slower cooling of the carcass at high outside temperatures (summer)	Original IML data	Ambient temperature at hunting day and duration between killing and evisceration affect IML	1
No or insufficient removal of e.g. hematomas, stomach and intestinal contents, adhering foreign materials (grass, leaves etc.)	Original IML data	Visible soiling affect IML	1
Cross-contamination of carcasses by e.g. other animals (stacking or too close placement of several killed animals on a transport vehicle) or due to insufficient hygienic conditions of the transport vehicle (e.g. soil, leaves, blood residues from eviscerated carcasses).	Experience reported by grey literature	-	4
